# Worldwide Genetic Variability of the Duffy Binding Protein: Insights into *Plasmodium vivax* Vaccine Development

**DOI:** 10.1371/journal.pone.0022944

**Published:** 2011-08-02

**Authors:** Taís Nóbrega de Sousa, Luzia Helena Carvalho, Cristiana Ferreira Alves de Brito

**Affiliations:** Laboratory of Malaria, Centro de Pesquisa Rene Rachou/Fiocruz, Belo Horizonte, Brazil; Federal University of São Paulo, Brazil

## Abstract

The dependence of *Plasmodium vivax* on invasion mediated by Duffy binding protein (DBP) makes this protein a prime candidate for development of a vaccine. However, the development of a DBP-based vaccine might be hampered by the high variability of the protein ligand (DBP_II_), known to bias the immune response toward a specific DBP variant. Here, the hypothesis being investigated is that the analysis of the worldwide DBP_II_ sequences will allow us to determine the minimum number of haplotypes (MNH) to be included in a DBP-based vaccine of broad coverage. For that, all DBP_II_ sequences available were compiled and MNH was based on the most frequent nonsynonymous single nucleotide polymorphisms, the majority mapped on B and T cell epitopes. A preliminary analysis of DBP_II_ genetic diversity from eight malaria-endemic countries estimated that a number between two to six DBP haplotypes (17 in total) would target at least 50% of parasite population circulating in each endemic region. Aiming to avoid region-specific haplotypes, we next analyzed the MNH that broadly cover worldwide parasite population. The results demonstrated that seven haplotypes would be required to cover around 60% of DBP_II_ sequences available. Trying to validate these selected haplotypes per country, we found that five out of the eight countries will be covered by the MNH (67% of parasite populations, range 48–84%). In addition, to identify related subgroups of DBP_II_ sequences we used a Bayesian clustering algorithm. The algorithm grouped all DBP_II_ sequences in six populations that were independent of geographic origin, with ancestral populations present in different proportions in each country. In conclusion, in this first attempt to undertake a global analysis about DBP_II_ variability, the results suggest that the development of DBP-based vaccine should consider multi-haplotype strategies; otherwise a putative *P. vivax* vaccine may not target some parasite populations.

## Introduction

After more than a century of development of malaria control measures, *Plasmodium vivax* remains more widely distributed than *Plasmodium falciparum* and it is a potential cause of morbidity and mortality. Around 2.85 billion people live at risk of infection by *P. vivax*, with the greatest burden occurring in Middle East, Asia, the Western Pacific, Central and South America [1], [2]. Although neglected, *P. vivax* causes important socioeconomic loss, with the overall global cost of *vivax* infection estimated as being between US$1.4–4.0 billion per year [3]. The recent emergence of drug-resistant strains and severe (sometimes fatal) disease challenges the traditional view of *P. vivax* malaria as a benign infection [4], [5]. Consequently, the malaria eradication research agenda (MalERA) placed the *P. vivax* in the top list of priorities [www.ploscollections.org/malERA2011].

The complex life cycle of the *Plasmodium* includes an erythrocytic phase that is responsible for clinical symptoms of human malaria. While *P. falciparum* invades mature as well immature red blood cells (RBC) through multiple invasion pathways, *P. vivax* invades preferentially reticulocytes [6] and requires mainly the interaction of parasite ligand to Duffy antigen/receptor for chemokines (DARC) on RBC membrane [7]. The *P. vivax* ligand is a 140 kDa micronemal type I membrane protein, called the Duffy binding protein (DBP), and gene-deletion experiment showed that DBP plays an important role in the irreversible junction of the merozoite with host erythrocytes, a key step of human infection [8]. Cysteine-rich region II of the DBP (DBP_II_) comprises erythrocyte binding motif known as Duffy-binding-like domain (DBL) [9], which is also found in other erythrocyte binding proteins (erythrocyte binding antigen 175 - EBA-175, EBA-140 and EBA-181) and in cytoadherent proteins (*Plasmodium falciparum* erythrocyte membrane protein 1) [10]. The crystal structure of the orthologous DBP ligand domain of the simian malaria *Plasmodium knowlesi* provided insight into the molecular basis for receptor recognition of the PvDBP. The proposed DARC-recognition site of DBP lies in a solvent-accessible groove on a fairly flat surface and exposed site for DARC recognition in subdomain 2 of DBP_II_ [11]. Recently, Bolton and Garry (2011) demonstrated an additional region on subdomain 1 of DBP_II_ that might be also necessary for DARC binding [12].

DBP_II_ is an important anti-*P.vivax* vaccine candidate since antibodies against this molecule: (i) inhibit *in vitro* their binding to DARC; (ii) reduce merozoite invasion of human erythrocytes; and (iii) confer protection against blood-stage infection [13], [14], [15], [16]. The important role of DBP-DARC interaction is reinforced by previous studies that showed individuals without DARC on their erythrocytes surface are highly resistant to *P. vivax* invasion [17], [18]. In addition, studies developed in Brazil and Papua New Guinea showed reduced susceptibility to *P. vivax* infection in heterozygous carriers of one DARC-negative allele compared to two DARC-positive allele carriers [19], [20]. However, the paradigm of the absolute dependence on the presence of Duffy on the red cell for *P. vivax* infection has been recently questioned for some findings indicating that *P. vivax* can infect and cause disease in Duffy-negative people [21], [22], [23]. Nevertheless, this situation seems to occur in specific areas and/or a small proportion of the populations, thus the epidemiological importance of this alternative pathway seem to be restricted to specific endemic areas, such as Madagascar [23].

Analysis of genetic variability from field parasites showed that the DBP binding domain (region II) is highly polymorphic [24], [25], [26], [27], [28], [29], [30], [31], therefore it might hamper the vaccine development as some variable residues alter immune recognition of protein [32], [33], [34]. The excess of non-synonymous substitutions observed in DBP_II_ is consistent with the hypothesis of positive selective pressure acting on this protein domain, and suggests allelic variation as a mechanism of immune evasion [35]. As antigenic variation presents a major limitation in successful vaccine design, we analyzed all DBP_II_ sequences recorded in GenBank in order to identify the main haplotypes shared among *P. vivax* isolates from different malaria-endemic areas and undertake a detailed analysis of the nucleotide diversity of the *dbpII* gene. Here we show the need to include a minimum number of DBP_II_ haplotypes in a DBP-based vaccine; otherwise no broad coverage against worldwide *P. vivax* isolates might be reached.

## Materials and Methods

### *P. vivax* isolates

The following 511 Duffy binding protein gene sequences of *P. vivax* deposited in GenBank (last update on 13th April 2011) were downloaded for genetic analyses: the sequence from a reference strain Sal-I (access number: NC_009911); 113 sequences from Papua New Guinea isolates (PNG) (DQ156519; Wosera area in East Sepik Province: AF289480–AF289483, AF289635–AF289653 and AF291096; Madang town and rural villages within Madang Province: AY970837–AY970925, AF469515–AF469602); 17 sequences from Colombia isolates (COL) obtained from the villages of Villavicencio and Tumaco (U50575–U50590, DQ156513); 15 sequences from South Korea isolates (SK) (DQ156515, DQ156522–DQ156523, AF215737–AF215738, AF220657, AF220659–AF220667); 102 sequences from different parts of India (IND) (FJ491142–FJ491241); 30 sequences from Thailand (THAI) (EF219451, EF368159–EF368180, EF379127–EF379132, EF379134); 100 sequences from Sri Lanka (SLK) (GU143914–GU144013); 123 sequences from different parts of Brazil (BRA) (DQ156520, EU812839–EU812960) and eleven sequences from Iran (IRA) (EU860428–EU860438). Only unique haplotypes for each individual were analyzed.

### Data Analysis

#### Genetic diversity analysis

DBP_II_ sequences were aligned and compared using the Clustal W multiple alignment algorithm in BioEdit Sequence Alignment editor [36] to identify the single nucleotide polymorphisms (SNPs). Gaps were removed from alignments because indels (insertions/deletions) and repeats evolve by different mechanisms than SNPs and might result in false estimates of biologically significant diversity. The number of segregating sites (S), haplotypes (H), nucleotide diversity (π), haplotype diversity (H*d*), and the corresponding standard deviations (SD) were estimated using DnaSP 5.10 software [37]. Between-population differentiation using the pairwise fixation index *F*_ST_ was measured with Arlequin 3.5 software [38]. *Haplotype construction*. Haplotypes (combinations of nucleotides with no particular weight placed upon any position) were constructed by using DnaSP 5.10. We removed synonymous SNPs to focus the analysis only on the protein diversity. *Cluster (population) analysis*. To determine whether our sample could be grouped into genetic clusters and to infer the number of clusters (*K*) that best fit the data, we used the Bayesian clustering method implemented in the Structure 2.3 software [39], [40]. Structure was run 10 times for *K* = 1–10 for 30,000 Monte Carlo Markov Chain (MCMC) iterations after burn-in period of 10,000 using the admixture model and correlated allele frequencies. We did not use prior information about population origin for each individual (USEPOPINFO = 0). The mean log probability of the data (Ln *P*[D]) and its standard deviation was plotted to predict the optimal value for *K*. Graphs of Structure results were produced by using the DISTRUCT program [41].

## Results

### Polymorphism and genetic differentiation

We compiled 511 sequences from GenBank for the gene fragment encoding Duffy binding protein region II (DBP_II_). The population dataset included sequences from the natural parasite populations of eight countries (Table 1): Brazil [35], Colombia [25], India and Iran [30], Papua New Guinea [27], [42], [43], South Korea [28], Sri Lanka [31] and Thailand [44]. The average of sample was 64 sequences (ranged from 11 to 123) per country. By analyzing a region of the DBP_II_ of 676 bp that is available for the overall dataset, 127 polymorphic sites were identified (ranged from 16 to 73 per country) with a nucleotide diversity (π) varying between 0.006 and 0.0109 (South Korea and Thailand, respectively). Most of these SNPs (55%) are singletons, i.e. observed only in one sequence. Despite the wide range of number of haplotypes per country (ranged from 9 to 73, mean of 24), the levels of haplotype diversity (Hd) among them were equally high and quite similar (ranged from 0.922 to 0.993 in Sri Lanka and Colombia, respectively). In order to remove most potential sequencing errors that could interfere with the analysis and interpretation of the results, additional analyses were performed excluding singleton polymorphisms (Table S1). By comparing both analyses a significant bias could be detected in the number of segregating sites and haplotypes in South Korea and PNG samples. However, nucleotide (π) and haplotype diversity (Hd) were not significantly affected by this further analysis because these diversity parameters exclude polymorphisms that are present at low frequencies.

**Table 1:** Estimates of diversity and genetic differentiation for PvDBP_II_ encoding gene among *P. vivax* isolates.

Population (N)	S	π (SD)	H	Hd (SD)	*F*_ST_						
					BRA	COL	PNG	SK	THAI	IRA	SLK
BRA (123)	21	0.0082 (0.0003)	35	0.935 (0.012)	-						
COL (17)	16	0.0085 (0.0007)	16	0.993 (0.023)	0.163*	-					
PNG (113)	73	0.0106 (0.0004)	73	0.981 (0.005)	0.117*	0.196*	-				
SK (15)	18	0.0060 (0.0013)	10	0.924 (0.053)	0.197*	0.384*	0.234*	-			
THAI (30)	29	0.0109 (0.0005)	24	0.982 (0.014)	0.083*	0.234*	0.127*	0.177*	-		
IRA (11)	17	0.0094 (0.0016)	9	0.964 (0.051)	−0.011	0.136*	0.063*	0.169*	0.040	-	
SLK (100)	27	0.0097 (0.0005)	39	0.922 (0.014)	0.029*	0.186*	0.129*	0.200*	0.101*	0.018	-
IND (102)	38	0.0088 (0.0005)	36	0.923 (0.016)	0.011*	0.183*	0.120*	0.200*	0.077*	0.004	0.018*
All (511)	127	0.0101 (0.0002)	193	0.970 (0.004)							

N: number of isolates; S: number of segregating sites; π: average number of nucleotide substitutions per 1000 sites between pairs of sequences (standard deviation, SD); Hd: haplotype diversity (SD); H: number of haplotypes; F_ST_: Fixation index, a measure of genetic differentiation between populations;

*: *F*_ST_ values with *P*<0.05. BRA – Brazil, COL – Colombia, IRA – Iran, SK – South Korea, SLK – Sri Lanka, THAI – Thailand, IND – India, PNG – Papua new Guinea.

To determine how the observed diversity was distributed among geographic regions, population structure was inferred by measuring genetic differentiation among countries (*F*_ST_). The highest differentiation was identified between Colombia and South Korea (*F*_ST_ = 0.384) and the lowest differentiation was detected between Iran and Brazil (*F*_ST_ = −0.011) (Table 1). We repeated the analyses for *F*_ST_ using the dataset in which singletons were excluded and no significant differences were observed for *F*_ST_ values among countries (Table S1).

### Haplotype diversity

To focus the analysis on the putative antigenic diversity (i.e. polymorphisms that change protein sequence), the nonsynonymous single nucleotide polymorphism (nsSNP) haplotypes were derived for DBP_II_ sequences. In total 46 nsSNPs were identified among all sequences with most of them being rare (19 nsSNPs with frequency ≤1% and 17 with frequency between 1–10%). Only 10 nsSNPs showed allele frequency above 10% in the whole world (R308S, K371E, G384D, E385K, K386N, H390R, N417K, L424I, W437R, and I503K). All but one nsSNP were found at the eight countries with DBP genetic diversity data available, except for the SNP R308S that was absent in Colombia and South Korea (Table 2). Moreover we investigated if these polymorphisms were localized in regions previously identified or predicted as T- or B-cell epitopes. All but one nsSNP (K371E) are in regions predicted or experimentally identified as epitope in the DBP_II_ (Table 2).

**Table 2:** Description of the most frequent nonsynonymous (nsSNPs) polymorphisms used to construct DBP_II_ haplotypes, their frequencies in each geographic population and presence in described epitopes.

nsSNP^a^	Frequency									
	BRA	COL	IRA	SK	SLK	THAI	IND	PNG	World	Epitope^b^
R308S	0.073	0.000	0.182	0.000	0.130	0.267	0.098	0.690	0.235	H1, 5
K371E	0.260	0.176	0.182	0.467	0.340	0.200	0.324	0.115	0.254	-
G384D	0.187	0.412	0.455	0.467	0.060	0.233	0.137	0.345	0.211	H3, 45
E385K	0.203	0.176	0.182	0.067	0.200	0.467	0.304	0.097	0.209	H3, 45, 48
K386N	0.228	0.176	0.182	0.067	0.200	0.400	0.304	0.097	0.211	H3, 45, 48
H390R	0.504	0.941	0.636	0.533	0.340	0.433	0.353	0.504	0.456	H3, 45, 48
N417K	0.398	0.412	0.364	0.933	0.360	0.400	0.373	0.336	0.387	M2, Ia *
L424I	0.480	0.412	0.545	1.000	0.490	0.867	0.451	0.681	0.558	M2, 66, Ia
W437R	0.488	0.118	0.455	1.000	0.370	0.633	0.373	0.327	0.417	M3 *
I503K	0.431	0.059	0.636	1.000	0.550	0.567	0.569	0.425	0.497	Id, 1638*

a : Amino acid numbers according to SAL-1 sequence [39] with the first letter representing the amino acid present in Sal-1 sequence and the latter representing the polymorphism;

b : H1, H3, M2 and M3 are B-cell epitopes [51]; 5 – T and B-cell epitope; 45 and 48 are B-cell epitopes; 66 – T-cell epitope [52], [53]; Ia and Id – MHC class I *in silico* predicted promiscuous epitopes [35]; 1638 – MHC class II universal epitope [54];

*- polymorphisms that alter the antigenic character of DBP_II_ [32].

**Figure 1:**
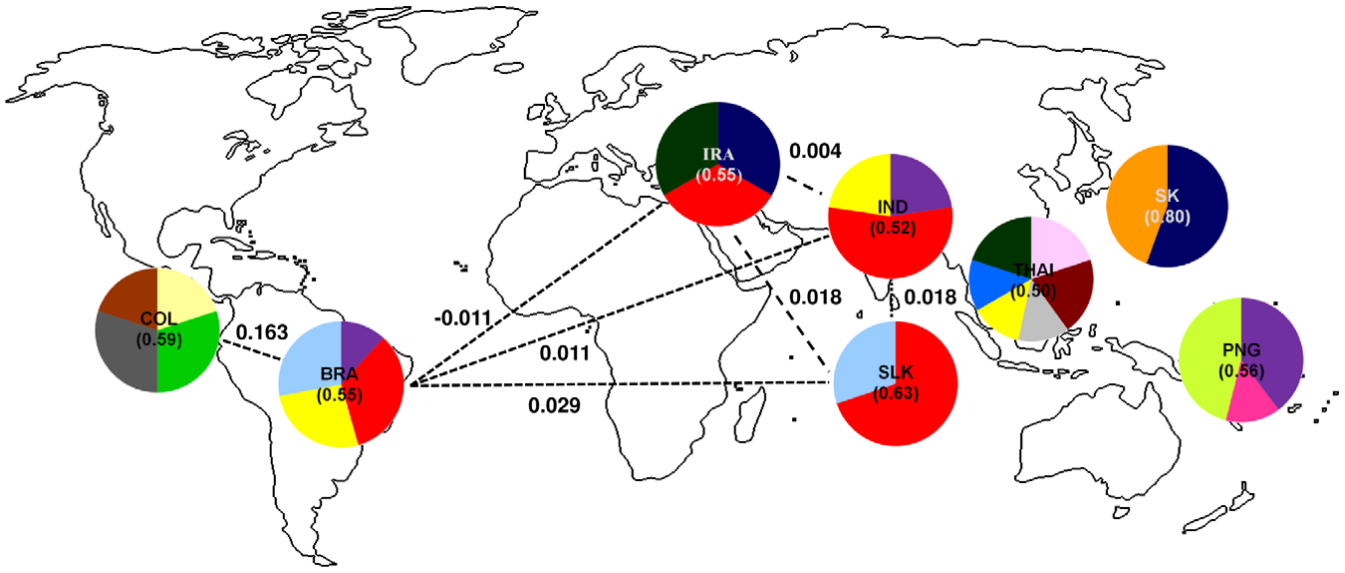
Frequency by country of the DBP_II_ haplotypes that cover at least fifty percent of local parasite population. Among the 73 haplotypes defined using the 10 most prevalent nonsynonymous single nucleotide polymorphisms (nsSNPs), 17 haplotypes (each haplotype was represented by a color) were able to cover at least 50% of each country parasite population: 2 in South Korea (Hap 4 – dark blue and Hap 40 - orange) and in Sri Lanka (Hap 23 - red and Hap 44 – light blue); 3 in Iran (Hap 4, Hap 23 and Hap 59 – dark green), in India (Hap 12 - purple, Hap 23 and Hap 24 - yellow) and in PNG (Hap 12, Hap 55 – pink and Hap 64 – light green); 4 in Colombia (Hap 9 – light yellow, Hap 27 – dark gray, Hap 30 - green and Hap 37 - brown) and 4 in Brazil (Hap 12, Hap 23, Hap 24 and Hap 44); 6 in Thailand (Hap 8 – dark brown, Hap 15 – light pink, Hap 21 - blue, Hap 24, Hap 36 – light gray and Hap 59). The haplotype from Sal-1 reference strain was represented by purple (Hap 12), for haplotype sequences please see Table S2. The number in parentheses indicates the frequency of the selected haplotypes in the respective country (range from 0.52 to 0.63). Some *F*_ST_ values among countries are showed and represented by dashed lines.

In further analysis, those 10 most frequent polymorphisms were used to build predominant DBP_II_ haplotypes circulating in malaria-endemic areas. Seventy-three haplotypes were defined for the whole dataset, ranging from 7 to 29 haplotypes per country (Table S2). In order to determine a minimum number of DBP_II_ haplotypes (MNH) required to be included in anti-*P. vivax* vaccine, we next sought to identify the number of haplotypes per country able to cover at least 50% of local parasite population. In the whole dataset, 17 out of 73 haplotypes fitted this criterion: two haplotypes in South Korea and Sri Lanka; three in Papua New Guinea, Iran and India; four haplotypes in Brazil and Colombia and six haplotypes in Thailand (Figure 1 and Table S2). Together, the 17 haplotypes covers about 70% of worldwide parasite population. Among them, seven were shared between two or more areas, being one haplotype (Hap 23, colored in red) found in high frequency in four countries from two different continents (America and Asia). This result agrees with those from *F*_ST_ analysis, which estimated low genetic differentiation between DBP_II_ sequences from Brazil and Iran, India or Sri Lanka (Table 1, Figure 1 and Table S2). The reference strain Sal-1 sequence, which has being used to develop a DBP_II_–based vaccine, covers only 10% of worldwide samples (Hap 12, colored in purple in Figure 1). So far, Sal-1 DBP_II_ was detected only in three endemic areas (Brazil, India and PNG), being in very low frequency in other countries (Figure 1 and Table S2).

**Figure 2:**
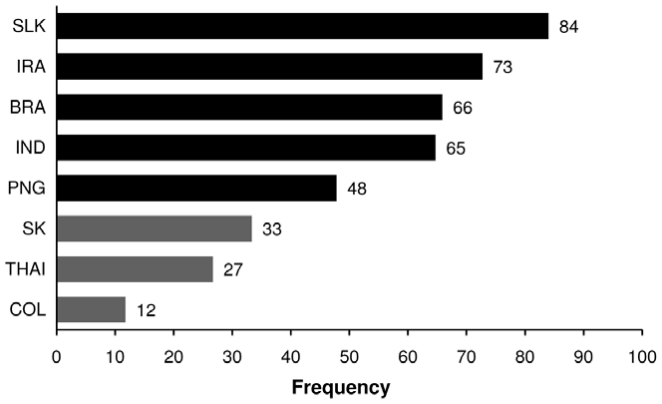
Frequency of the seven nsSNP haplotypes of DBP_II_ that cover at least fifty percent of DBP_II_ sequences deposited in GenBank. Coverage of the selected haplotypes in each country: countries that at least 50% of parasites are covered by the selected haplotypes are represented by black bars and countries that less than 50% of the parasites are covered by the selected haplotypes are represented by grey bars. The selected haplotypes were: 4, 12, 23, 24, 44, 59, 64 (for haplotype sequences see Table S2).

Concerning a more global approach for DBP-based vaccine, we further sought to determine the MNH that will be able to cover the majority (∼50%) of worldwide parasite population independent of the region of origin. Seven haplotypes fitted this criterion and were found in 60% of 511 DBP_II_ sequences of *P. vivax* deposited in GenBank (Haplotypes 4, 12, 23, 24, 44, 59, 64 of Table S2). Considering the distribution of these 7 selected haplotypes by locality, it was possible to categorize those localities in two groups (Figure 2). The first group includes Sri Lanka, Iran, Brazil, India and PNG, where around 50% of the parasites population will be covered by these haplotypes. The second group includes South Korea, Thailand and Colombia, where about only 24% (range 12–33%) of parasite isolates will be covered by these seven selected haplotypes.

### Clustering

**Figure 3:**
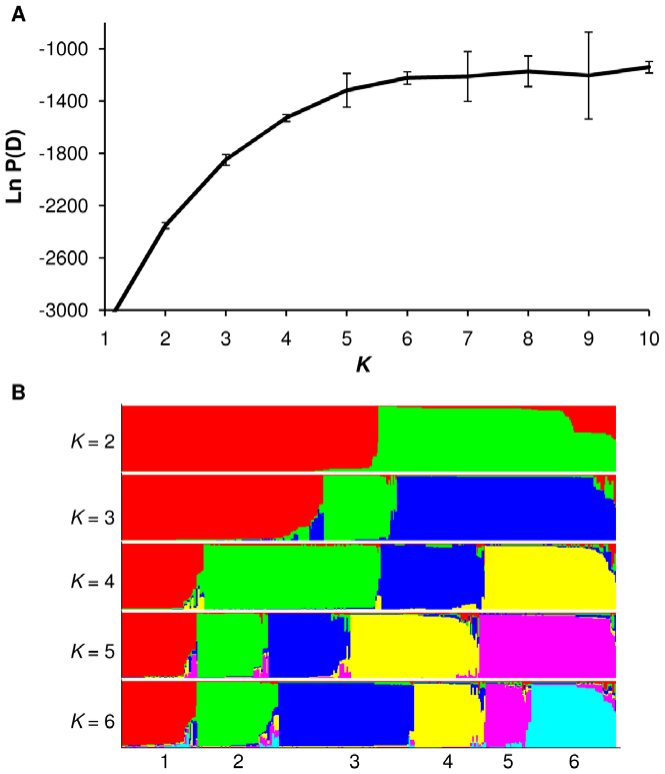
Population structure of the gene encoding DBP_II_ based on Bayesian cluster analysis using Structure 2.3 software. (A) Plot of the log probability of the data [Ln *P*(D)] and its standard deviation (vertical bars) given values for *K* of 1–10. (B) Population clustering for *K* = 2–6. Each individual is represented by a thin vertical color. Each color represents an ancestral population (pop), and the color of individual represents their proportional membership in the different populations. Ancestral populations: red, pop1; green, pop2; blue, pop3; yellow, pop4; pink, pop5 and light blue, pop6. The figure shown for a given *K* is based on the highest probability run at that *K*.

**Figure 4:**
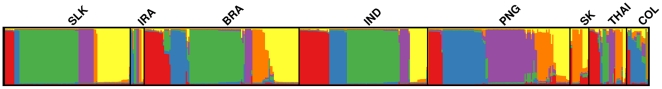
Population structure for *P. vivax* sequences of DBP_II_ at *K* = 6. Graphic of Structure results were produced by using the DISTRUCT program. Individual *P. vivax* isolate are represented by a thin vertical line from each geographic population showed above the graphic and separated by a thin black line.

Aiming to identify related subgroups of DBP_II_ sequences among parasites circulating in the study countries, individual samples were clustered to population on the basis of their genotypes, independent of their geographic location. For that, we used the clustering method that uses departures from Hardy-Weinberg equilibrium to detect population structure. The algorithm groups related individuals into a predefined number of clusters (*K*), herein *K* = 1–10. A Bayesian approach is taken to infer the *K* value that provides the best fit to the data as measured by the log-likelihood score. Each individual is then assigned a membership coefficient (*Q*) to each of the clusters with majority of the haplotypes being assigned to only one cluster at “true” *K*. The estimated log probability of our data [Ln *P*(D)] plateaued between *K* = 4–6 (Figure 3A). Simulation studies have shown that once the real *K* has been reached, Ln *P*(D) will typically plateau or continue to increase slightly, indicating that *K* = 6 provides the best fit to our data. We show clustering results for *K* values of 2–6, being each individual represented by a vertical line, and each ancestral population in a different color (Figure 3B). To determine whether the above-defined subgroups were geographically restricted we plotted the average *Q* for each country. For all countries but South Korea the analysis supported low levels of differentiation among geographical regions, with three to six ancestral populations present in each country (Figure 4).

## Discussion

The major pathway used by *P. vivax* to invade human reticulocytes depends on the interaction of the DBP with its cognate receptor, which makes DBP a high priority anti-*P. vivax* vaccine candidate. A significant challenge for the development of DBP-based vaccines is its highly polymorphic nature, which can biases antibodies response toward a specific DBP variant [32], [33], [34]. Engineering vaccines that combine all potential haplotypes that might be circulating in malaria-endemic areas could circumvent polymorphism's limitation; however, it is not feasible for a highly polymorphic antigen like DBP. Therefore, the purpose of this study was to identify predominant DBP_II_ haplotypes that could be included in a putative vaccine, with potential to induce an immune response against *P. vivax* circulating around the world.

By comparing the DBP_II_ diversity found in different countries worldwide, we demonstrated high levels of haplotype diversity among *P. vivax* isolates. The profile of DBP_II_ genetic diversity was not related with the levels of malaria endemicity, since similar pattern was observed from areas with low and unstable malaria transmission, such as Brazil, as well as from highly endemic areas such as Papua New Guinea. These findings suggested that recombination plays an important role in determining the haplotype structure of DBP_II_, as we recently demonstrated [35]. Genetic recombination of parasites takes place in the vector, as part of the *Plasmodium* life cycle, and is likely facilitated by multiplicity of infections, i.e. the simultaneous infection of a host by more than one parasite variant. For *P. falciparum*, the levels of multiplicity of infection are partially correlated with the levels of transmission intensities [45]. Nevertheless for *P. vivax*, strikingly high values of multiplicity of infection are reported even in regions of low endemicity such as Brazil and Thailand. Thus, the proportion of multiple-clone *P. vivax* infections, estimated by using microsatellites analyses, range from 49–57% in Brazil [46], [47], 10–47% in India [48], [49], 9–60% in Sri Lanka [50] and 52–63% in Thailand [48], [49]. This difference in pattern of multiplicity of infections between *P. vivax* and *P. falciparum* could be related with specific biological features of the *P. vivax* parasite, such as earlier gametocytogenesis [51] and relapse [52]. Early gametocytogenesis might allow for a more efficient transmission to the mosquito vector before symptoms appear and, thus, before drug treatment is initiated, while relapses would also enhance transmission and increase the probability of detecting mixed infections as further inoculations occur.

Besides the role of recombination we have recently demonstrated that natural selection has an important role in the generation and maintenance of the genetic diversity of DBP_II_ [35]. The maintenance of this variability by diversifying selection presumably helps parasite to evade the host immune recognition and favors a low-medium frequency of distinct haplotypes. As we can expect due differences in the endemicity spectrum and immune response profile, both recombination and selection seem to be acting differentially among distinct geographical areas. If no recombination is assumed, we would expect that the number of described polymorphic sites observed would be arranged into a maximum of n+1 haplotypes. Here, this profile was found in the majority of studied countries, some of them with the number of haplotypes lower than the number of segregating sites (South Korea and Iran). However, it is important to highlight that the small number of DBP_II_ sequences available for those two countries might biased the number of haplotypes observed for those regions; specially, because there was a significant correlation between the number of haplotypes and sample size (Spearman correlation coefficient: r_s_ = 0.6628, P = 0.0139). Interestingly, in two countries, Brazil and Sri Lanka, the number of haplotypes was higher than the number of segregating sites, suggesting a major role of recombination in these areas. At this time, it is not possible to conclude if the differences on haplotype number could be consequence of different recombination rates or due to the different levels of selective pressure.

For the purpose of rationalizing vaccine design will be necessary to define polymorphisms that represent antigenically distinct haplotypes. Here, we selected the most frequent nonsynonymous single nucleotide polymorphisms (nsSNPs) to derive DBP_II_ haplotypes. Nine out of 10 nsSNPs lay in regions of DBP_II_ that are immunologically relevant, mapping on previously defined T- and B-cell epitopes [32], [35], [53], [54], [55], [56]. Based on these nsSNPs, we identified a number between two and six DBP_II_ haplotypes, which will be required to cover 50% of parasite population in each studied country. Unfortunately, most of those haplotypes were geographically restricted and we could not find a single high-frequency haplotype covering all endemic areas. Of note, the DBP_II_ Sal-1 variant that is currently being used to develop a DBP_II_–based vaccine was found so far in low frequency (10%) and it seems to be restricted to specific geographic areas. Aiming to avoid geographically restrict DBP_II_ haplotypes, in the next analysis we determined the MNH that covers at least fifty percent of DBP_II_ sequences available in GenBank independent of the region of origin. By using this approach, seven frequent haplotypes were identified, that broadly cover parasite populations from 5 out of 8 endemic-countries, i.e., Sri Lanka, Iran, Brazil, India and PNG. These same selected haplotypes were not able to cover parasite populations from South Korea, Colombia and Thailand; however, the results for South Korea and Colombia must be interpreted with caution because of the limited number of sequences available. Together, these results show that the development of DBP-based vaccine should considered multi-haplotype strategies. Similar strategy has been proposed to another polymorphic malaria vaccine candidate, the *P. falciparum* Apical Membrane Antigen 1 (PfAMA-1) [57]. By using a similar approach to that used here, the authors demonstrated that a worldwide collection of PfAMA1 haplotypes could be clustered into six populations that were independent of geographic location. Because continuous culture of *P. falciparum* has been successfully automated for over 30 years [58], the same authors were able to demonstrate that immunization with one member of PfAMA-1 elicited antibodies that block *in vitro* parasite invasion against the same subgroup [57]. Altogether, these two studies provide an important proof of concept that vaccine against malaria polymorphic antigens should consider multi-haplotype strategies. Now it remains to be determined whether those DBP_II_ haplotypes described here could elicit broadly immune response. Although essential, those studies will be a difficult task due to limitation on *P. vivax* culture. Recent progress in short-term culture of *P. vivax* field isolates [59], [60] and *in vitro* DBP_II_-DARC binding assays [15], [61] present new opportunities to improve understanding of the ability of DBP_II_ haplotypes to elicit cross-reactive responses against those that are genetically similar. In addition, these assays might help to define which polymorphisms determine antigenically different DBP_II_ haplotypes.

An additional consideration in the malaria vaccines design is the geographic structuring of the parasite populations because significant variation among regions would suggest a need for vaccines to be tailored accordingly. Overall, we detected low to medium differentiation between countries, except to sequences from Colombia and South Korea, which showed high differentiation compared to the other regions. Remarkably, the four haplotypes that covers more than half Colombian parasites were not shared with any other parasite population, even with the Brazilian, another Latin America parasite population. The contrast among-region structuring was confirmed by *F*_ST_ analysis with whole data set and clustering analysis using the STRUCTURE software with the ten selected nsSNPs. The Bayesian clustering tool identified six subgroups of related DBP_II_ sequences in the worldwide parasite population. Nevertheless, clustering was not related to the geographical origin of *P. vivax* isolates and all subgroups were present in the eight endemic countries studied, although in different proportions. Moreover, the large majority of isolates (>75%) was formed predominantly by only one ancestral population. Similar pattern was described for *P. falciparum* merozoite antigens in a meta-population analysis [62]. In that study, the merozoite antigens generally had lower levels of differentiation among countries. In addition, cluster analysis suggested that haplotypes formed subgroups independent of geographic origin with high levels of diversity within population. Thus, to improve probability of broad efficacy, vaccines based on merozoite antigens as DBP_II_ may incorporate predominant haplotypes from different regions. Further analysis will be required to define the stability of the haplotypes in a region over time, as natural fluctuations can result from frequency dependent selection or new haplotypes can appear as result of recombination. For DBP, a single study so far has investigated the dynamic of asymptomatic *P. vivax* infections in a cohort of PNG children [63]. By following those children over six-month period, it was possible to demonstrate that the DBP_II_ dominant haplotypes remained relatively stable throughout the study. However, it is difficult to determine the period of time necessary to observe such changes.

The present study comprises the first attempt to undertake a global analysis about DBP_II_ variability and provides new insights on future design of a broad-spectrum *P. vivax* vaccine. Overcome the limitation of DBP diversity may require the inclusion of representative haplotypes otherwise a large proportion of the *P. vivax* population will be not target.

## Supporting Information

Table S1 Estimates of genetic diversity and differentiation for PvDBP_II_ encoding gene among *P. vivax* isolates in the absence of singleton sequences.
(DOC)

Table S2 Characterization of DBP_II_ haplotypes present in the eight endemic countries studied.
(DOC)
