# Supplementary material for: Worldwide Genetic Variability of the Duffy Binding Protein: Insights into Plasmodium vivax Vaccine Development
Source: PLoS One. 2011 Aug 2;6(8):e22944. doi: 10.1371/journal.pone.0022944 (PMC3149059; doi:10.1371/journal.pone.0022944)
Supplement: Table S1 — Estimates of genetic diversity and differentiation for PvDBPII encoding gene among P. vivax isolates in the absence of singleton sequences. (DOC) [file pone.0022944.s001.doc]

**Table S1. Estimates of genetic diversity and differentiation for PvDBP_II_ encoding gene among *P. vivax* isolates in the absence of singleton sequences**

| **Population (N)** | **S** | **π (SD)** | **H** | **Hd (SD)** | ***F*_ST_** | | | | | | |
| --- | --- | --- | --- | --- | --- | --- | --- | --- | --- | --- | --- |
|  |  |  |  |  | **BRA** | **COL** | **PNG** | **SK** | **THAI** | **IRA** | **SLK** |
| BRA (123) | 20 | 0.0092 (0.0004) | 34 | 0.934 (0.012) | - |  |  |  |  |  |  |
| COL (17) | 15 | 0.0093 (0.0007) | 16 | 0.993 (0.023) | 0.168* | - |  |  |  |  |  |
| PNG (113) | 30 | 0.0106 (0.0004) | 44 | 0.940 (0.012) | 0.126* | 0.223* | - |  |  |  |  |
| SK (15) | 9 | 0.0047 (0.0007) | 7 | 0.838 (0.068) | 0.194* | 0.424* | 0.255* | - |  |  |  |
|  |  |  |  |  |  |  |  |  |  |  |  |
| THAI (30) | 23 | 0.0115 (0.0006) | 23 | 0.979 (0.014) | 0.087* | 0.256* | 0.145* | 0.192* | - |  |  |
|  |  |  |  |  |  |  |  |  |  |  |  |
| IRA (11) | 17 | 0.0104 (0.0018) | 9 | 0.964 (0.051) | -0.013 | 0.140* | 0.072* | 0.194* | 0.045 | - |  |
|  |  |  |  |  |  |  |  |  |  |  |  |
| SLK (100) | 25 | 0.0108 (0.0006) | 38 | 0.919 (0.015) | 0.029* | 0.189* | 0.136* | 0.196* | 0.105* | 0.017 | - |
| IND (102) | 29 | 0.0095 (0.0005) | 32 | 0.916 (0.017) | 0.012* | 0.194* | 0.131* | 0.201* | 0.081* | 0.002 | 0.017* |
| All (511) | 57 | 0.0109 (0.0002) | 149 | 0.964 (0.004) |  |  |  |  |  |  |  |
|  |  |  |  |  |  |  |  |  |  |  |  |

N = number of isolates; S = number of segregating sites; π = average number of nucleotide substitutions per 1000 sites between pairs of sequences (and SD); *Hd* = haplotype diversity (and its standard deviation. SD); H = number of haplotypes; *F*_ST_ = Fixation index, a measure of genetic differentiation between populations; * – *F*_ST_ values with *P*<0.05.
