# Supplementary material for: Worldwide Genetic Variability of the Duffy Binding Protein: Insights into Plasmodium vivax Vaccine Development
Source: PLoS One. 2011 Aug 2;6(8):e22944. doi: 10.1371/journal.pone.0022944 (PMC3149059; doi:10.1371/journal.pone.0022944)
Supplement: Table S2 — Characterization of DBPII haplotypes present in the eight endemic countries studied. (DOC) [file pone.0022944.s002.doc]

**Table S2**. Characterization of DBP_II_ haplotypes present in the eight countries studied.

| **Code** | **Haplotype^a^** | **Number of haplotypes^b^** | | | | | | | | **Total number of haplotypes^c^** |
| --- | --- | --- | --- | --- | --- | --- | --- | --- | --- | --- |
|  |  | **BRA** | **COL** | **IRA** | **SK** | **PNG** | **SLK** | **THAI** | **IND** |  |
| **1** | GAAAGGAACA |  |  |  | 1 |  |  |  |  | 1 |
| **2** | GAAAGGTACA |  |  |  |  |  |  | 1 |  | 1 |
| **3** | GAAGGAAACA |  |  |  |  | 1 |  |  |  | 1 |
| **4** | GAAGGGAACA | 8 |  | 2 | 5 |  | 2 | 1 |  | 18 |
| **5** | GAAGGGAACT | 2 | 1 |  |  | 1 |  | 1 |  | 5 |
| **6** | GAAGGGATTA |  | 1 |  |  |  |  |  |  | 1 |
| **7** | GAAGGGATTT |  | 2 |  |  |  |  |  |  | 2 |
| **8** | GAAGGGTACA |  |  |  |  |  |  | 3 |  | 3 |
| **9** | GAAGGGTATT |  | 2 |  |  |  |  | 1 |  | 3 |
| **10** | GAAGGGTTCA |  |  | 1 |  |  |  |  |  | 1 |
| **11** | GAAGGGTTTA | 4 |  | 1 |  | 6 |  |  | 2 | 13 |
| **12** | GAAGGGTTTT | 8 | 1 | 1 |  | 25 | 4 |  | 12 | 51 |
| **13** | GAAGTGAACA |  |  |  | 1 |  |  |  |  | 1 |
| **14** | GAAGTGTTCA | 1 |  |  |  |  |  |  |  | 1 |
| **15** | GAGATAAACA | 2 |  |  |  |  |  | 3 | 2 | 7 |
| **16** | GAGATAAACT | 5 |  |  |  |  |  |  |  | 5 |
| **17** | GAGATATATT | 1 |  |  |  |  |  |  |  | 1 |
| **18** | GAGATATTCA | 4 |  |  |  |  |  |  |  | 4 |
| **19** | GAGATGAACA |  |  |  |  |  |  |  | 1 | 1 |
| **20** | GAGATGAACT | 1 |  |  |  |  |  |  |  | 1 |
| **21** | GAGGGATATA |  |  |  |  |  |  | 2 | 1 | 3 |
| **22** | GAGGGATATT |  |  |  |  | 1 |  |  | 1 | 2 |
| **23** | GAGGGATTTA | 23 |  | 2 |  |  | 44 | 1 | 29 | 99 |
| **24** | GAGGGATTTT | 18 | 1 |  |  |  | 3 | 2 | 12 | 36 |
| **25** | GAGGGGAACA |  |  |  | 1 |  | 1 |  |  | 2 |
| **26** | GAGGGGAACT | 3 |  |  |  |  |  |  |  | 3 |
| **27** | GAGGGGTATT | 1 | 3 |  |  |  |  | 1 |  | 5 |
| **28** | GAGGGGTTCT | 1 |  |  |  |  |  |  |  | 1 |
| **29** | GAGGGGTTTA | 1 |  |  |  |  |  |  |  | 1 |
| **30** | GAGGGGTTTT |  | 3 |  |  |  |  |  |  | 3 |
| **31** | GAGGTAAACA | 1 |  |  |  |  |  |  |  | 1 |
| **32** | GAGGTGTTCA | 1 |  |  |  |  |  |  |  | 1 |
| **33** | GGGATAAACA |  |  |  |  |  |  |  | 7 | 7 |
| **34** | GGGATAAACT |  |  |  |  |  | 7 |  | 5 | 12 |
| **35** | GGGATGAACA | 3 |  |  |  |  |  |  | 5 | 8 |
| **36** | GGGATGAACT | 2 | 1 |  |  |  |  | 2 | 1 | 6 |
| **37** | GGGATGATTT |  | 2 |  |  |  |  |  |  | 2 |
| **38** | GGGATGTACT |  |  |  |  |  |  | 1 |  | 1 |
| **39** | GGGGAAAACA |  |  |  | 2 |  |  |  |  | 2 |
| **40** | GGGGGAAACA |  |  |  | 4 |  |  | 1 |  | 5 |
| **41** | GGGGGACACA |  |  |  | 1 |  |  |  |  | 1 |
| **42** | GGGGGATTTT | 1 |  |  |  |  |  |  |  | 1 |
| **43** | GGGGGGAACA |  |  | 1 |  | 1 | 6 | 1 | 7 | 16 |
| **44** | GGGGGGAACT | 19 |  | 1 |  |  | 19 | 1 | 6 | 46 |
| **45** | GGGGGGATCA |  |  |  |  |  |  |  | 1 | 1 |
| **46** | GGGGGGTACA | 2 |  |  |  |  | 1 |  |  | 3 |
| **47** | GGGGGGTACT | 1 |  |  |  |  |  |  |  | 1 |
| **48** | GGGGGGTTCT | 1 |  |  |  |  |  |  |  | 1 |
| **49** | TAAGGATACA |  |  |  |  | 1 |  |  |  | 1 |
| **50** | TAAGGGAACA |  |  |  |  | 1 |  |  |  | 1 |
| **51** | TAAGGGTACA |  |  |  |  | 1 |  |  |  | 1 |
| **52** | TAAGGGTTTA |  |  |  |  | 2 |  |  |  | 2 |
| **53** | TAAGGGTTTT |  |  |  |  | 1 |  |  |  | 1 |
| **54** | TAGAAAAACA |  |  |  |  |  |  | 1 |  | 1 |
| **55** | TAGATAAACA |  |  |  |  | 9 |  | 1 | 2 | 12 |
| **56** | TAGATAAACT |  |  |  |  | 1 |  |  |  | 1 |
| **57** | TAGATAAATA |  |  |  |  | 1 |  |  |  | 1 |
| **58** | TAGATATACT |  |  |  |  |  |  | 1 |  | 1 |
| **59** | TAGATATATT | 5 |  | 2 |  |  | 12 | 3 | 7 | 29 |
| **60** | TAGATATTCA |  |  |  |  |  |  | 1 |  | 1 |
| **61** | TAGGGAAACA |  |  |  |  | 2 |  |  |  | 2 |
| **62** | TAGGGAAATT |  |  |  |  | 2 |  |  |  | 2 |
| **63** | TAGGGATATA |  |  |  |  | 5 |  | 1 |  | 6 |
| **64** | TAGGGATATT |  |  |  |  | 29 |  |  |  | 29 |
| **65** | TAGGGATTTA | 1 |  |  |  |  |  |  |  | 1 |
| **66** | TAGGGATTTT |  |  |  |  | 2 |  |  |  | 2 |
| **67** | TAGGGGAACA |  |  |  |  | 6 |  |  |  | 6 |
| **68** | TAGGGGAACT |  |  |  |  | 1 |  |  |  | 1 |
| **69** | TAGGGGTATA |  |  |  |  | 2 |  |  |  | 2 |
| **70** | TGGATGAACA | 2 |  |  |  |  | 1 |  | 1 | 4 |
| **71** | TGGGGAAACA |  |  |  |  | 2 |  |  |  | 2 |
| **72** | TGGGGGAACA |  |  |  |  | 8 |  |  |  | 8 |
| **73** | TGGGGGAACT | 1 |  |  |  | 2 |  |  |  | 3 |
| **Total samples** |  | **123** | **17** | **11** | **15** | **113** | **100** | **30** | **102** | **511** |

^a^: Haplotype sequence based on the 10 nonsynonymous single nucleotide polymorphisms: nt 924 (R308S), 1111 (K371E), 1151 (G384D), 1153 (E385K), 1158 (K386N), 1169 (H390R), 1251 (N417K), 1270 (L424I), 1309 (W437R), and 1508 (I503K); ^b^: Absolute number of haplotypes per geographical region; c: Total number of haplotypes in the whole sample.
